# Atypical gunshot injury to the right side of the face with the bullet lodged in the carotid sheath: a case report

**DOI:** 10.1186/1752-1947-8-29

**Published:** 2014-01-27

**Authors:** Peter A Ongom, Stephen C Kijjambu, Josephat Jombwe

**Affiliations:** 1 Department of Surgery, School of Medicine, Makerere College of Health Sciences, Makerere University, P O Box 7072 Kampala, Uganda; 2 Department of Surgery, Mulago National Referral and Teaching Hospital, P O Box 7051 Kampala, Uganda

**Keywords:** AK-47 rifle, Carotid sheath, Facial injuries, Mandibular fracture, Middle ear injury, Nerve damage, Surgical extraction, Wandering bullet

## Abstract

**Introduction:**

Gunshot injuries of the head and neck from the AK-47 rifle (a common assault rifle, submachine gun type) are a significant contributor to morbidity and mortality among civilians in Sub-Saharan Africa. They may cause significant damage to the closely arranged structures in this region, and the bullet’s trajectory can be very difficult to determine. We present an unusual case of gunshot injury with an atypical bullet entry wound, profound injury to the face, lodgment in the right carotid sheath, and 'wandering’; a first of its kind in East Africa.

**Case presentation:**

A 27-year-old African-Ugandan woman of Nilotic ethnicity was referred to the Accident and Emergency Department of a tertiary hospital in Uganda, having sustained complex injuries due to an inadvertent AK-47 rifle gunshot injury. The gunshot injury was to the right side of her face with a large ragged entry wound and no exit wound. Prior basic wound care and radiological imaging showed a comminuted fracture of her mandible with lodgment of the bullet in her neck, anterior to her sixth and seventh cervical vertebrae. Standard debridement of her wound was done. A computed tomography scan showed an apparent cephalad shift ('wandering’) of the bullet, leaving it lying partially anterior to her fifth cervical vertebra as well as within her carotid sheath. Other injuries were to her facial and trigeminal nerves, and her middle ear. The 'wandering’ bullet was successfully removed surgically. It had caused no damage to any part of her neck structure.

**Conclusion:**

AK-47 rifle bullet injuries may present with uncharacteristically large entry wounds and cause complex structural injuries at the area of impact. The consequent trajectory is difficult to predict making regional examination and radiological investigations essential in management. Bullets may be retained, leaving no exit wound. Securing the airway, controlling hemorrhage and identifying other injuries are the first vital steps. This case illustrates all these interventions and the important decision to extract the entrapped bullet from the patient’s neck because it had started to 'wander’ and could have caused grave injury over time with further migration. Maxillofacial, plastic, trauma, general and military surgeons, otorhinolaryngologists and emergency physicians can gain from this experience because it calls for a multidisciplinary team approach.

## Introduction

Gunshot injuries (GSIs) cause profound morbidity and significant mortality [1]. Those involving the head and neck can be devastating especially when they affect vital organs. They can result in instant death. Facial GSIs can present as unusual and complicated clinical entities. Similar injuries occur in both military and civilian settings. Some geopolitical conflict areas in Africa have GSI as the second most common cause of death [2]. There is an increasing incidence of GSIs worldwide, particularly those involving the face [3]. The extent of damage is dependent on a number of factors, such as: magnitude of energy transferred, distance travelled by the missile, type of bullet, and the structures encountered before and on penetration. In general, high-energy transfer gunshots fired at close range inflict the most damage [4]. A classic weapon inflicting this mode of injury is the AK-47 rifle (a common assault rifle, submachine gun type) [5]. The extent of tissue damage depends on: internal lacerations, compression of tissues and the temporary cavitation along the projectile path [6]. Secondary injuries are also possible following impact with bone, which sets other missiles (bone fragments) into motion on their own paths, causing injury independent of the primary insulting missile [7].

The face and neck region is packed with vital structures in a relatively small volume of space. Even the smallest of movements by a penetrating missile may injure a major vein, artery and main nerve trunk simultaneously. The leading cause of death in penetrating neck trauma is major vascular injury leading to uncontrollable hemorrhage [8]. Treatment is challenging especially when the bullet or its fragments are lodged within the vicinity of vital structures [4]. Most bullets or their fragments are highly contaminated and pose a therapeutic dilemma to the surgeon especially when there is associated tissue loss. Tissue damage is both direct and as a result of energy dissipated from the inherent kinetic energy transferred to the tissues.

Missile injuries are broadly described as penetrating (25%), perforating (38%) and avulsing (37%) [4]. Some gunshot wounds are through-and-through injuries, but in many patients the bullet enters with no visible exit wound. In such situations, the bullet's trajectory and final destination may be unpredictable; there may be an uncertain extent of bony damage and unforeseen consequences to the patient's airway from hematoma or edema.

The ideal time and method of treatment remains a constant issue of debate [9,10]. Several surgeons argue that because of the mechanism of injury, early aggressive primary reconstruction might not be ideal. They opt for initial conservative management followed by a staged secondary reconstruction to obtain satisfactory functional and esthetic results [9]. Alternatively, others advocate early management of facial deformity [10]. It is with this perspective that surgeons universally come to some form of consensus regarding the management of GSIs to the face and neck. The steps taken include: securing the airway; controlling hemorrhage; identifying other injuries and preventing additional injury; and, repair and/or reconstruction of the traumatic facial deformities [11].

Bullets lodged in the neck have a tendency to migrate and wander away from their initial location. The period and path taken varies widely. Previous studies have shown embolization to the heart’s right atrium and ventricle [12], pulmonary artery [13], and through the left brachiocephalic vein to the right ventricle [14]. There may also be aspiration of the bullet into the larynx following initial impact on the mandible [15]. Each of these studies offers a unique description of the bullet’s trajectory and sequelae. Surgical removal has been necessary several years after the original injury in some instances [16].

Essential evaluation encompasses investigation for vascular injuries; the commonest threat to life. There is increased interest and growing experience in the use of non-invasive imaging techniques [17]. Contrast material-enhanced helical computed tomography (CT) angiography is increasingly being used to evaluate trauma patients in stable condition who are at risk of vascular injuries. It facilitates detailed characterization of traumatic vascular lesions in the neck. These may include: partial or complete occlusion, pseudo-aneurysm, intimal flap, dissection, and arteriovenous fistula. In the same setting, CT angiography provides valuable additional information about the cervical soft tissues, the airway, the digestive tract, the spinal canal and the spinal cord. In cases of penetrating GSIs, the trajectory of the bullet and the locations of fragments can be assessed.

## Case presentation

A 27-year-old African-Ugandan woman of Nilotic ethnicity presented to the Accident and Emergency Department of a tertiary hospital, following a GSI to her face. She was brought as a referral from a regional hospital, 300km away. The injury was the result of a 'stray’ bullet, shot from an AK-47 submachine gun at a distance of about 70m. At that time, she experienced intense pain over the right side of her face, collapsed and passed out for about 20 minutes. The amount and type of blood loss (arterial versus venous) was difficult to ascertain. She was rushed to the local regional hospital after bystanders had applied improvised bleeding-control measures (wrapping the head with clothing). Resuscitation and a surgical wound toilet were done without extension of the wound. Essential antibiotics and vaccines were administered, and the wound was dressed (not sutured). Injuries had been sustained over the right side of her face, leaving a large open wound.

**Figure 1:**
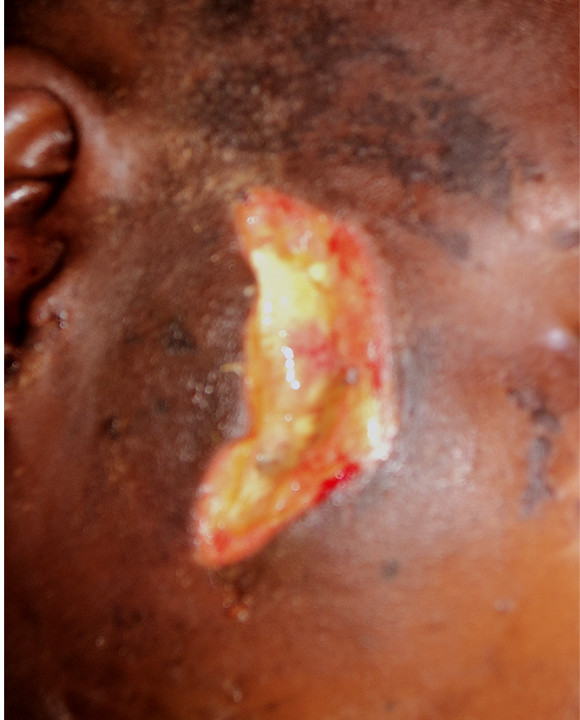
**Photograph showing an atypical bullet entry wound.** Appearance of the wound 10 days after injury. There is evidence of suppuration and areas of granulation tissue formation.

She was referred to the tertiary hospital 10 days after these events. Her main complaints were right mandibular pain, with difficulty in opening her mouth and chewing, and inability to hear with her right ear. She was fully conscious and was hemodynamically stable but had moderate pallor. There was a laceration of her maxillomandibular area (6cm in length) with features of infected granulation tissue (Figure 1). The right side of her maxillofacial region was swollen and tender. There was no active bleeding, subcutaneous emphysema, or dyspnea and stridor. She had only a very limited capacity to open her jaw, and even then with pain. There was no particularly painful area in her neck. Remarkable findings were revealed on cranial nerve examination. She had a right facial nerve palsy (lower motor neuron) and total loss of cutaneous sensation over the distribution of the right maxillary and mandibular branches of her trigeminal nerve in the maxillary and mandibular regions respectively. The ophthalmic division of her trigeminal nerve was intact. Her auditory function was assessed. A Rinne’s test revealed conduction deafness of her right ear; a Weber’s test showed that her auditory nerves were intact. Sensation over her neck was intact as well. Superficial temporal artery pulsation was present.

**Figure 2:**
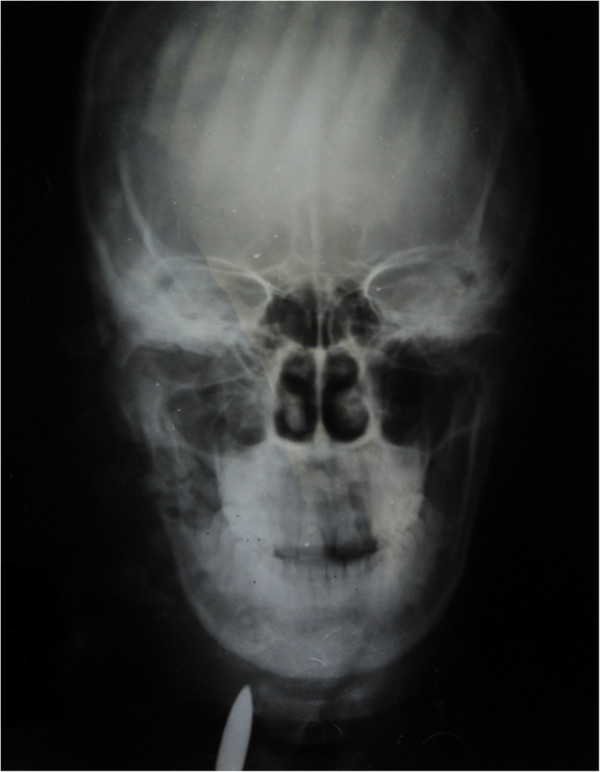
**Anteroposterior radiograph of the head and neck.** This shows a bullet in the right side of the neck. Comminuted fracture of the mandible (angle and ramus) and maxillary antrum are revealed.

**Figure 3:**
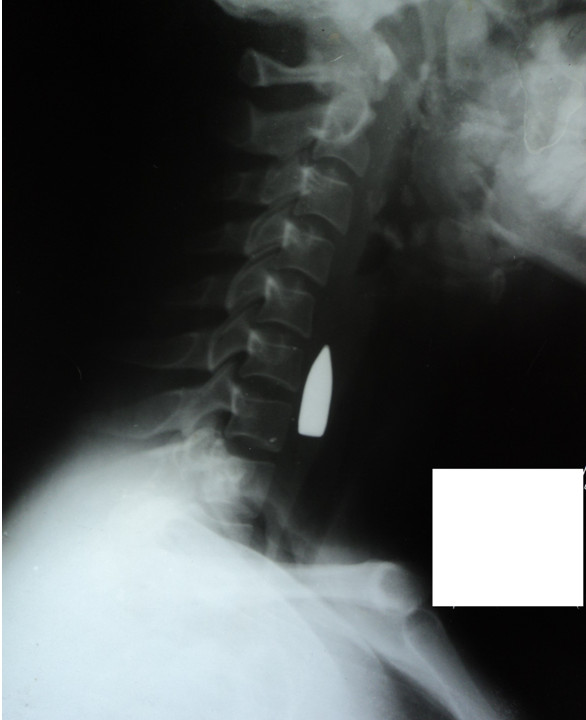
**Lateral radiograph of the head and neck.** This shows a bullet lodged in the neck, anterior to the bodies of cervical vertebrae 6 and 7.

A plain radiograph of her head and neck (Figures 2 and 3) and an ultrasound scan of her neck, done prior to her referral, had revealed the presence of a bullet in the right side of her neck, anterior to cervical vertebrae 6 and 7. Basic as it is, this plain radiograph enabled identification of this important condition. It also revealed comminuted fracture of her mandible (angle and ramus) and maxillary antrum.

Subsequent management at our unit proceeded with exploration of the bullet entry wound. A comminuted fracture of the ramus and angle of her right mandible was confirmed. Debridement was done and the entry wound was closed. Fracture fixation was not done due to logistical obstacles. She was put on a course of parenteral antibiotics: clindamycin, 150mg, three times a day; ceftriaxone, 1g, twice a day, for 5 days. Analgesics (parenteral diclofenac, 75mg, three times a day; and morphine, 5mg, four times a day) were also administered.

**Figure 4:**
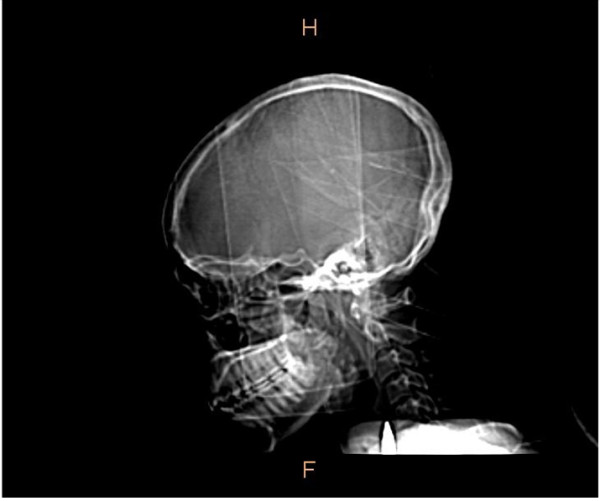
**Computed tomography scan of the head and neck.** The scan shows a cephalad shift of the bullet to lie partially anterior to the fifth cervical vertebra. There is also a comminuted fracture of the angle and ramus of the right mandible. The section is through the median sagittal plane.

The management plan was then focused on full establishment of the bullet’s trajectory with the aim of its extraction as deemed essential at this point in time, given the migratory tendency of foreign bodies (missiles) in the neck and subsequent vascular injury. Further evaluation involved conducting a CT scan of her head and neck 1 week after admission. This confirmed the earlier radiological findings; a bullet not only lodged in the right side of her neck but within her carotid sheath as well (Figure 4). The CT scan however, showed that it was no longer positioned just anterior to cervical vertebrae 6 and 7, but also anterior to cervical vertebra 5; a shift in the cephalad direction ('wandering’ or 'migration’) from what the previous radiographs showed. This could be a real shift or an apparent one, due to some cervical flexion during the CT procedure. It was lateral to her trachea, posterolateral to her common carotid artery, and just posterior and intimate with her internal jugular vein, within her carotid sheath.

**Figure 5:**
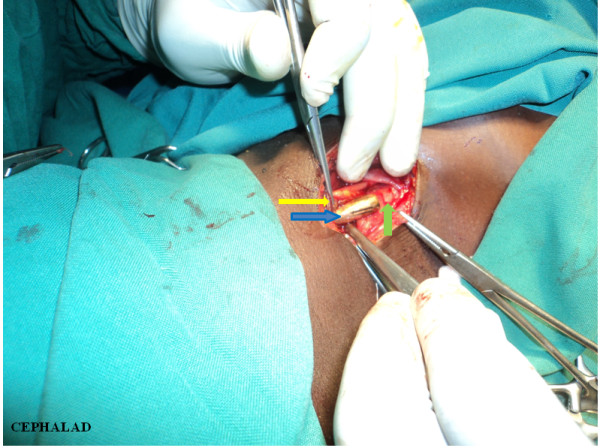
**Photograph of bullet extraction.** The right carotid sheath is opened exposing the retained bullet (blue arrow). Artery forceps grasp the open carotid sheath (yellow arrow). An intact area of the carotid sheath covering the caudal end of the bullet (green arrow).

**Figure 6:**
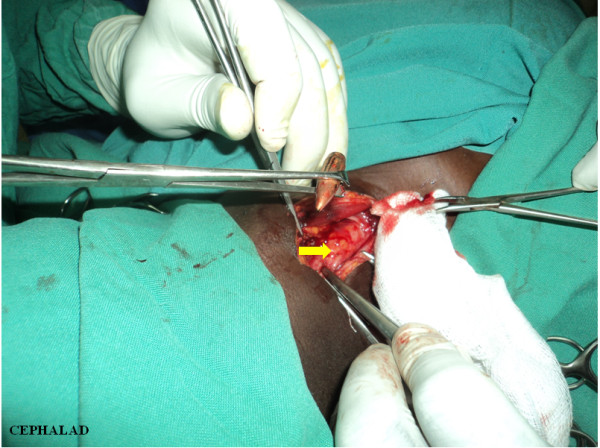
**Photograph following bullet extraction.** The bullet had lodged in the carotid sheath with damage neither to the common carotid artery (yellow arrow) nor to the internal jugular vein. There is no suppuration.

A decision to remove the bullet operatively was made in view of the 'wandering’ scenario. A right longitudinal cervical incision, just posterior and parallel to her sternocleidomastoid muscle, was used to access the posterior triangle of her neck. The investing deep fascia was dissected and her carotid sheath opened. The bullet was identified, confined within a pocket of debris (Figure 5). There were no signs of suppuration, and acute inflammation had resolved. The debris possibly consisted of damaged tissue encountered along the trajectory of the bullet: parotid gland tissue, blood, nerve and muscle remnants. The bullet was extracted, debris was flushed out thoroughly with normal saline (50mL) and her sheath was closed with Vicryl 2–0 continuous suture (Figure 6). Subcutaneous sutures (Vicryl 3/0, interrupted) were placed. Her postoperative recovery was uneventful.

The way forward then was: to fix the mandibular fracture; explore the possibilities of nerve repair and facial/mastication muscle re-establishment of function; and to conduct a full auditory workup to assess the extent of damage with the aim of restoration of function or rehabilitation. Unfortunately, due to socioeconomic reasons, she has not yet had this done and has had to leave hospital with the following injuries still outstanding: auditory conduction damage; facial, mandibular and maxillary nerve damage; and, mandibular and maxillary antrum fractures.

## Discussion

The case presents us with a clinical 'syndrome’ as a result of an AK-47 GSI. The entire extent of such injuries can be difficult to identify, and removal of the lodged bullet in the cranio-maxillofacial area to prevent secondary complications and reduce the chance of secondary infection can be more complicated than in other parts of the body [5]. Injuries are typically high-energy transfer in nature and the bullet is potentially lethal within a range of up to 200m. Our patient was well within this range. The entire collection of her individual injuries illustrates the extent of complications after penetrating mandibular injury. A strategy for management of such an occurrence is also contained in the clinical workup she received [15].

We were faced with a scenario of a GSI to the right side of her head. Given the distance of about 70m, we expected substantial injury; a GSI to the head is often immediately fatal. It was a penetrating type of injury, although avulsion per se was also possible [4]; a 6cm-long wound. What is not clear concerning the mechanism is the nature of motion, velocity and energy of the bullet at the time of impact with the face. Damage inflicted is proportional to the energy transferred from the missile to the tissues. The following are the possible characteristics of the bullet at impact: a direct bullet 'hit’ , a ricocheting bullet (indirect), or a 'falling’ bullet. More specifically a direct 'hit’ could be either 'head-on’ or 'broadside-on’ , whereas a ricocheting bullet would have come off another object, with partial loss of energy depending on the density of the object encountered first. The patient’s history does not make this any clearer. Bullet entry wounds generally have a particular appearance, including contusion, skin introflection, and simple or excoriated ecchymosis. The skin wound is typically a hole with frayed margins. A 'head-on’ direct 'hit’ would be expected to have an entry wound that is small, with a shape and diameter of the missile [6], whereas a 'broadside-on’ direct 'hit’ tends to present a more ragged and irregular pattern. These are basically high-energy transfer injuries. A 'falling’ bullet would be low-energy transfer in nature and have any size and shape of entry wound [5]. It is also unlikely to have been fired from a distance of only about 70m. The damage it causes is limited to the influence of gravity on it. A bullet having ricocheted off another object first, would be subjected to all the secondary movements of a missile: 'twisting’ , 'yawing’ , 'wobbling’ and 'tumbling’. This will cause an atypical entry wound. The injury we are dealing with seems to have been the result of a ricocheting bullet.

At the first health facility, measures were taken to secure her airway, control hemorrhage and identify other injuries, through the radiological investigations [11]. This is the first and most important step in the patient’s management. Some gunshot wounds are through-and-through injuries, but in many patients the bullet enters with no visible exit wound [4]. Among these individuals, the bullet's trajectory and final destination are very important in the extent of bony damage and injury to the airway and vascular structures. Moreover the head and neck is a relatively small body part in volume with several vital structures closely packed. Even a small distance traveled can mean damage to a number of nerves, blood vessels and bone, among others. In this case there is a comminuted fracture of her mandible and maxillary antrum; severance of her facial nerve, mandibular nerve, and maxillary nerve; and damage to her middle ear.

The bullet, having lodged in her neck, had typically started migrating in a cephalad direction. Within a period of less than 2 weeks, it had shifted position from lying anterior to the cervical vertebrae 6 and 7 and was now lying anterior to vertebrae 6 and 7, as well as vertebra 5. This is a significant shift given the size of the neck in perspective of the vital structures it contains. An ultrasound scan and CT scan had also shown the bullet’s intimacy with her common carotid artery and internal jugular vein; it was therefore an immediate danger to them. Migration in this case could lead to: arteriovenous fistula formation, vascular perforation with resultant exsanguination, embolization to her heart through her internal jugular vein, and a wandering bullet in her mediastinum. It is important to examine all structures surrounding the trajectory/pathway of the bullet for any secondary injuries following the fracture. The physical examination and radiological tests facilitated this. Bone fragments themselves set off extra missiles which can have an entirely different pathway [7]. Her nerve and bone injuries were a direct effect of the bullet at entry. The damage to her middle ear is more plausibly explained by the transmission of force through her temporomandibular joint following impact with the angle and ramus of her mandible.

Surgical extraction of the bullet was essential and took priority in this case because it had started wandering and posed an immediate danger to vital structures. In the event that there was no bullet migration, and the patient had presented later, removal could have been delayed but may still be necessary even several years later [17]. Priority would then have been given to mandibular fracture fixation, auditory damage repair, and neuron repair. A good guiding principle is that a bullet ceases to cause damage when it ceases to move [18].

## Conclusions

Our case demonstrates a situation involving both direct and indirect high-energy transfer injury: a hallmark of the AK-47 rifle. It also represents a penetrating form of GSI with no exit point. More conspicuous is an atypical entry wound and an unusual carotid sheath lodgment. The bullet ostensibly having started 'wandering’ from its initial position called for surgical extraction within 3 weeks of the initial insult.

We draw important inferences. Firstly, it is essential that we follow the lines of basic management of facial and neck injuries, that is: securing the airway; controlling hemorrhage; identifying other injuries and preventing additional injury; and, repair of the traumatic facial deformities, in that order. Secondly, local/regional radiological investigations (plain radiographs and CT scans) are vital tools in assessing collateral damage and whether the bullet has begun to 'wander’. Thirdly, bullets may lodge in an unusual site (carotid sheath) calling for surgical extraction quite early in the course of management. In this case it ought to be done prior to definitive repair and rehabilitation of damage to the ear, nerves and mandible. Finally, entry wounds may be atypical, giving complicated missile trajectories. Ultimately though, bullet wounds always require a multidisciplinary team approach to management.

## Consent

Written informed consent was obtained from the patient for publication of this case report and all accompanying images. A copy of the written consent is available for review by the Editor-in-Chief of this journal.

## Abbreviations

CT: Computed tomography; GSI: Gunshot injury.

## Competing interests

The authors declare that they have no competing interests.

## Authors’ contributions

PAO conceptualized the idea, wrote the manuscript, performed surgery and managed the patient; SCK conducted scientific editing and critical review of the manuscript for intellectual content; JJ co-conceptualized the idea, performed surgery and managed the patient. All authors read and approved the final manuscript.
